# If you build it, will they come? Exploring the success factors of knowledge management systems in the Malaysian public sector

**DOI:** 10.1016/j.heliyon.2024.e27093

**Published:** 2024-03-07

**Authors:** Nor'ashikin Ali, Musyazid Md Mustaffa, Gamal Alkawsi, Luiz Fernando Capretz

**Affiliations:** a College of Computing & Informatics, Universiti Tenaga Nasional, Malaysia; b Institute of Informatics and Computing in Energy, Universiti Tenaga Nasional, 43000, Selangor, Malaysia; c Faculty of Computer Science and Information Systems, Thamar University, Yemen; d Department of Electrical and Computer Engineering, Western University, London, ON N6A 5B9, Canada

**Keywords:** Knowledge management systems, Information systems success, Public sector, Malaysia, Organizational factors, Organizational culture

## Abstract

The current study investigates the factors that influence the success of knowledge management systems in the public sector. This study integrates the DeLone and McLean Model with critical organizational factors. The model has been tested on the data collected from 158 employees in the public sector in Malaysia, the study found that knowledge content quality has a higher significant impact on the use of knowledge management systems than system quality. Perceived usefulness also has a greater impact than user satisfaction in determining the system's overall success. Among the organizational factors, leadership is the most significant determinant of success. However, the culture of sharing, perceived trust, and incentives do not significantly influence the use of knowledge management systems. The findings suggest that public sector organizations should focus on both system and organizational factors to implement successful knowledge management systems.

## 1 Introduction

In transition countries, Knowledge Management (KM) plays a pivotal role, underscoring the critical importance of effective knowledge management for ensuring the sustained competitiveness of organizations [1]. Despite being predicted to be a management trend decades ago, KM is not dead. A previous study provided evidence that KM has made significant progress and is still “alive and kicking” [2]. With the increasing use of the Internet and the rising dependence on digital technologies in organizations, more data are processed, and more information is used; thus, more knowledge needs to be managed to create value for organizations. In the modern competitive business environment, where the organization's value relies on its knowledge assets, managing and sharing knowledge is essential. To ensure that KM continues to contribute to the sustainable development of organizations, it is important to understand what organizations should do for successful KM.

In practicing KM, many researchers have stressed the potential benefits of Information and Communication Technology (ICT), most notably in facilitating KM processes such as knowledge repository and knowledge transfer. While KM is not directly connected to technology, implementing KM requires ICT to enable KM initiatives to be performed efficiently. In the literature, ICT applications identified as having been linked with KM are email, online discussion forums and knowledge portals, databases, social media, and audio or video conferencing [[3], [4], [5]]. These applications enable knowledge sharing among individuals in organizations; these are the platforms through which they communicate, provide feedback, and discuss their ideas.

Siami-Namini (2018) [6], highlighted that KM has been adopted by both the private and public sectors for competitive advantage and sustainability. In the public sector, with its specialized workforce and responsibility for critical services, effective KM is essential for preserving and utilizing employee knowledge to enhance decision-making and innovation.

Like any other type of information system, KMS has a high chance of failure if there are no strategies to determine its success. The public sector has a long history of failed ICT projects [[7], [8], [9], [10]], and thus, this should be a lesson learned for KMS implementation. Previous studies of ICT and KMS have for some time emphasized the importance of understanding success factors to improve the success of any ICT implementation, including KMS [[11], [12], [13], [14]]. Although KM has for some time been one of the components of organizations’ strategies [13], there are still limited studies of KMS success in the public sector, and not much is known about what contributes to KMS success in the public sector.

Previous studies associated KMS success with the willingness of individuals to use KMS to share knowledge [15,16]. Regardless of the sophisticated KMS, it still fails to entice employees to contribute to the system. One of the reasons found in the literature is a lack of factors that motivate employees to share knowledge. Motivation is a necessary prerequisite for employees to use KMS to share their knowledge [[17], [18], [19]]. Since knowledge resides in people, it cannot be effectively shared unless those people are motivated to share it. There have been numerous reported cases of KM initiative failure due to employees’ reluctance to share their knowledge, although the KMS is available as a platform for them to contribute their knowledge. Therefore, evaluating KMS success should include not only technical factors but also organizational factors. While technical factors can solve system issues, organizational factors can solve human issues. Although numerous studies of KMS success are growing, there are still limited empirical studies on KMS success that involve both technical and organizational factors in determining KMS success in the public sector. Further analyzing these factors could have great significance for the public sector. The objective of this study is to identify factors that determine the success of KMS implementation in the public sector. This study focuses on the role of organizational factors in addition to technical factors, as previous studies have shown that focusing on technical factors is still ineffective in determining the success of KMS implementation.

In conclusion, the objective of this study is to identify factors that determine the success of KM system implementation in the public sector. From the main research goal, two research objectives for this study are as follows: (1) to determine the system factors that influence the success of KM systems in the public sector; and (2) to determine the organizational factors that influence the success of KM systems in the public sector.

The remainder of this study is organized as follows. Section 2 reviews the related work. Section 3 explains the theoretical concept. Section 4 assesses the research methodology. Section 5 summarizes the findings of the analysis. Section 6 discusses the results of the analysis. Section 7 summarizes the implications of the findings. Finally, Section 8 concludes the article.

## 2 Literature review

In general, public or private organizations have adopted the concept of KM, but it is more widely spread in the private sector. For instance, in Malaysia, the Malaysian Administrative Modernization and Management Planning Unit (MAMPU) and the Malaysian Agricultural Research and Development Institute (MARDI) are two public sectors that have implemented KM [[20], [21], [22]]. However, compared to private sector organizations, most private agencies have implemented KM, such as Bank Negara Malaysia, Petronas, Tenaga Nasional Berhad, Telekom, and Multimedia Super Corridor (MSC) companies [23,24]. It shows that the public sector is still far behind the private sector in KM implementation.

Various studies have been conducted to determine KM practices and challenges in the public sector. For instance, Ismyrlis (2020)[25] stated that KM challenges to the public sector in Greece are the lack of monetary rewards for knowledge transfer, the individualistic attitudes of public sector employees, and the failure to embrace ICT practices and facilitate electronic government. Organizational characteristics in the public sector are sophisticated. They are historically isolated, with well-defined networks comprising formal hierarchies and systematic structural components as well as a variety of informal principles. Currently, public sector organizations are characterized by a blend of structured hierarchical characteristics, formal decision-making processes, and tightly controlled bureaucratic accountability structures [26]. According to Khan et al. (2021) [27], recognizing KM as a discipline in the public sector is difficult due to the different environments of public service delivery and the outcomes of service orientation aspects that complicate policy implementation.

Laihonen and Mäntylä (2018) [28], conducted a case study to overcome challenges in leveraging performance information by implementing KM for the local government in Tampere, Finland. The authors suggested the following four critical factors for the success of strategic KM in local government: strategic priority, KM integration into management systems, data refinement, and the quality of data. The findings also highlighted the importance of management culture and continuous performance communication within the organization. In Slovenian public sector organizations, Colnar and Dimovski (2017) [29], stated that one of the most difficult challenges is changing the organizational culture or perception of employees. Other challenges include poor management of information technology, unmotivated employees, and the perception of domestic business organizations as a primarily bureaucratic system. In the Malaysian public sector, the findings from the survey conducted by MAMPU show that KMS in the public sector is not fully optimized due to a lack of sharing culture and different interpretations of the knowledge management concept [20]. Implementing KMS initiatives would foster a culture of knowledge sharing and provide support in allocating resources for knowledge investment.

Previous studies have found a variety of challenges to knowledge sharing and transfer within an organization. Lack of trust has been proven to be the most crucial barrier that prevents knowledge sharing in organizations [[30], [31], [32]]. KM practitioners should foster a culture that promotes employee trust and recognizes them for taking on knowledge initiatives in the public sector. Another barrier to KMS implementation is the significance of an incentive or motivation for the employee. When employees are not motivated to share their knowledge and there is no reward for doing so, they prefer to conceal their abilities and not share them with others [31]. Conversely, if an organization rewards an employee for sharing knowledge, the employee becomes more motivated to share knowledge, which results in organizational learning within public sector organizations.

Leadership is critical in promoting knowledge sharing and transfer to successfully achieve sustainable benefits within public sector organizations. Employees can be encouraged to learn and adapt new and existing knowledge to gain a competitive advantage through KM methods if top management supports, trusts, and recognizes them. A closed-minded employee and management style can be a challenge to KM, which results in a decrease in overall innovation capability within the organization [33]. Successful KM practice necessitates an open culture that promotes knowledge sharing, while individual qualities have an important impact on cultural transformation. A previous study stated that the lack of a sharing culture among organizational members might hinder KM practices [34]. In the public sector, some employees are hesitant to obtain information and knowledge from their colleagues.

Similarly, employees who are less open to diversity avoid knowledge sharing and transfer. Furthermore, a lack of communication has been recognized as critical to knowledge sharing and transfer within an organization [35]. Siami-Namini (2018) [6] claimed that the retirement of public servants and the regular knowledge exchange among government employees present new challenges for knowledge retention, institutional memory preservation, and the training of new staff [6]. Capturing tacit knowledge and then training staff is critical so that it can be passed on to new employees. However, knowledge can be effectively stored with the advancement of ICT infrastructure. Stored knowledge can be shared using the appropriate platform [34]. The quality of knowledge content and KM systems must be updated regularly with scheduled maintenance to ensure that relevant information and knowledge reach the appropriate employee.

## 3 Hypothesis development and conceptual framework

### 3.1 Knowledge content quality

High knowledge content quality is essential in the public sector because it influences top management actions, which can seriously affect their decision-making process. Employees tend to recognize the usefulness of a KMS implementation if it is perceived as informative, relevant, correct, and timely in decision-making and problem-solving. When knowledge is widely accessible, its availability for performing tasks is perceived as useful [36]. When knowledge content is of high quality, employees are more likely to believe that KMS will help them improve their job performance, influencing their decision to use KMS to increase task productivity. From the perspective of the preceding discussion, the following hypotheses are addressed.

H1 A higher degree of knowledge content quality leads to a higher degree of perceived usefulness for KMS.

H2 A higher degree of knowledge content quality leads to a higher degree of user satisfaction.

### 3.2 KM system quality

Previous studies have confirmed the influence of KMS system quality on user satisfaction [[11], [12], [30], [37]]. Employees tend to use a system if they feel it is effective and would benefit their job performance. Therefore, the organization must pay attention to updating the system technology regularly and allocate adequate human resources, especially the IT officer in charge, in case of any KMS problems. Any vulnerabilities in low or high system quality that affect employees with greater difficulty are likely to impact the public sector significantly. From the perspective of the preceding discussion, the following hypotheses are addressed.

H3 A higher degree of KM system quality leads to a higher degree of perceived usefulness for KMS.

H4 A higher degree of KM system quality leads to a higher degree of user satisfaction.

### 3.3 Perceived usefulness of KMS

Previous studies have shown that perceived usefulness impacts user satisfaction [[11], [12], [37], [38]]. Employees may discover value in using and sharing knowledge if they believe that KMS not only contributes to their work outcomes but also helps to minimize the additional effort required to share, retrieve, and use knowledge. The perceived usefulness of KMS can help employees work together more rapidly to navigate difficult situations and share their knowledge. If employees believe that KMS will be useful, they are more likely to use it. In addition, if KMS is perceived as useful, it is more probable that it will satisfy the employees’ requirements, which makes them more satisfied with KMS. From the perspective of the preceding discussion, the following hypotheses are addressed.

H5 A higher degree of perceived usefulness leads to a higher degree of KMS use.

H6 A higher degree of perceived usefulness for KMS leads to a higher degree of user satisfaction.

### 3.4 User satisfaction

Researchers have highlighted the effect of user satisfaction on KMS use in the context of KMS success. User satisfaction has been shown to significantly impact KMS use in previous studies [[11], [30], [37]], while Kulkarni, Ravindran and Freeze (2006) discovered that user satisfaction influences knowledge use. If employees are satisfied with the capabilities of KMS, they are more likely to use it. Employees will effectively use KMS if they understand that it can help them improve their job performance [12]. Otherwise, they will not contribute to using KMS if it does not provide benefits. From the perspective of the preceding discussion, the following hypothesis is addressed.

H7 A higher degree of user satisfaction leads to a higher degree of KMS use.

### 3.5 Culture of sharing

Ali, Cob and Sulaiman (2016) conducted a study to determine KMS success in the context of higher education institutions in Malaysia. The authors suggested that the sharing culture plays a vital role in influencing academicians’ perceptions of the usefulness of KMS [39]. In contrast, Chen et al. (2012) [40], in their case study of electronic manufacturing firms in Taiwan, found that organizational climate significantly influences knowledge sharing among employees when they work in a trusted, supportive, and pro-sharing environment. This study assumes that because the use of KMS is an option, the culture of sharing is expected to influence the perspective of using KMS rather than directly influencing behavior. From the perspective of the preceding discussion, the following hypothesis is addressed.

H8 A culture of sharing leads to a higher degree of perceived usefulness for KMS.

### 3.6 Perceived trust

Most previous studies on the influence of perceived trust on KMS success are insufficient. In the context of KMS success, He, Fang and Wei (2009) [32] surveyed a global IT corporation in China. The authors discovered that in KMS, perceived trust significantly impacted the perceived usefulness of knowledge-seeking. Nevertheless, Astuti and Suryadi (2015) [30] did not find any correlation between trust and perceived usefulness. Based on the responses, the authors found that the users trust the knowledge in KMS but do not trust everything within KMS. This study assumes that trust beliefs will enable employees to form opinions about KMS use, thereby determining their influence on quality perceptions of KMS usefulness. From the perspective of the preceding discussion, the following hypothesis is addressed.

H9 A higher degree of perceived trust leads to a higher degree of perceived usefulness for KMS.

### 3.7 Leadership

In their study of higher education institutions, Ali, Cob and Sulaiman (2016) [39] confirmed that leadership affects the quality of knowledge content and the use of KMS. Kulkarni, Ravindran and Freeze (2006) Kulkarni [12], found in their study of KMS success in corporate organizations that leadership affects KMS use and knowledge content quality. The authors asserted that integrating leaders in promoting knowledge sharing will encourage employees to submit high-quality knowledge and use KMS knowledge. Employees are more likely to appreciate the system's usefulness in an organizational environment that provides explicit and considerable leadership influence for KMS implementation. Taking into account the preceding discussion, the following hypotheses are addressed.

H10 A higher degree of leadership leads to a higher degree of knowledge content quality.

H11 A higher degree of leadership leads to a higher degree of KMS use.

### 3.8 Incentive

Ali et al. (2017)[37] found that incentives influence the use of KMS for sharing but have no effect on the quality of knowledge content. This means that tangible incentives are more important for increasing the quantity of commitments, but not necessarily the quality. In another study, Kulkarni, Ravindran and Freeze (2006) [12], discovered that knowledge use and knowledge content quality are influenced by incentives. This suggests that if employees are rewarded for sharing knowledge, they are more likely to do so with higher-quality knowledge. The top leaders of the organization should make it clear that they support the KMS as well as improve reward systems that allow users of the system to experience real benefits and that employees believe that their use of the system is valuable, justified, and essential. From the perspective of the preceding discussion, the following hypothesis is addressed.

H12 *A higher degree of incentive leads to a higher degree of knowledge content quality*.

### 3.9 Research model

A conceptual model for measuring KMS success in the public sector is developed by combining system and organizational factors, and their relationships are examined.

The DeLone and McLean model served as the foundational framework for our research for several compelling reasons. First, this model is widely recognized and has been extensively used to assess the success of information systems, providing a well-established foundation for our study. Second, the model's dimensions of system quality, information quality, service quality, and user satisfaction align closely with our research focus on system factors such as knowledge content quality, perceived usefulness, and user satisfaction.

Our investigation of the DeLone and McLean model specifically catered to the context of KMS in the public sector. In our model, we adapted and included additional constructs to address the nuances of KMS in a public sector setting. By doing so, we aimed to create a model that not only draws from established IS success theory but also considers the unique characteristics and requirements of KMS implementation in the public sector.

**Fig. 1:**
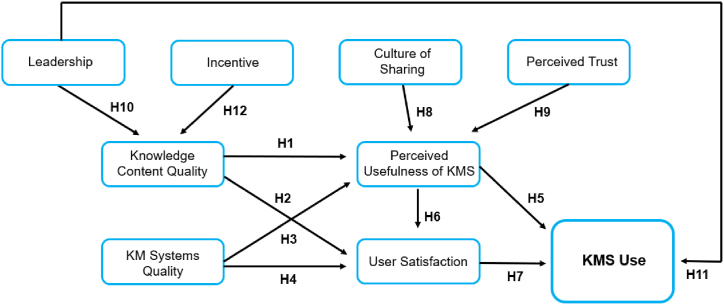
Conceptual model of KMS success in the public sector.

Fig. 1 illustrates the conceptual model of KMS success in the public sector. The conceptual model adopts system factors, including knowledge content quality, KM system quality, perceived usefulness of KMS, and user satisfaction, and organizational factors, including the culture of sharing, perceived trust, leadership, and incentive. This study assumes that KMS success is related to the utilization of ICT tools by employees within public sector organizations. Therefore, the present study adopts KMS use as the outcome variable for KMS success.

## 4 Method

A well-structured research methodology is crucial for ensuring the reliability, validity, and rigor of our study. In this section, we provide an overview of the research design, data collection, measurement instrument, and data analysis techniques employed to achieve the objectives of our study. Our research methodology is driven by the need to explore the complex interplay of system and organizational factors influencing KMS success in the unique context of the public sector.

In this study, we employed a comprehensive methodology to investigate the determinants of KMS success in the Malaysian public sector. Our study used a cross-sectional survey to collect data from employees across various public-sector organizations. We designed a structured questionnaire, encompassing system factors (e.g., knowledge content quality, perceived usefulness, and user satisfaction) and organizational factors (e.g., leadership and culture of sharing) relevant to KMS success. Partial Least Squares Structural Equation Modeling (PLS-SEM) was employed for analyzing data, assessing complex relationships between variables, and validating hypotheses. The methodology employed in this study aimed to provide a robust foundation for understanding the intricate factors shaping KMS success in the public sector and offering practical insights for organizational improvement.

The following subsections will provide detailed insights into each aspect of our research methodology, beginning with an explanation of the research design and followed by discussions on data collection, instrument development, data analysis, and hypothesis testing.

### 4.1 Survey design

The literature contains acceptable quality measures, and items from the current measurements have been reused. The relevant items were drawn from multiple studies when constructing the measurements of the construct to ensure the content fits in with the domain of KMS success in public sector areas [[11], [12], [32], [37], [41], [42]]. To make each measure suitable for the context of the study, items were examined and reworded when necessary. If more than one measure was available for a given construction, the best content fit was selected as the basis.

To receive feedback on the entire structure of the questionnaire, a group of government officers with information systems backgrounds were selected to pretest the questionnaire. Respondents were instructed to first complete the instrument before providing input on concerns such as format, content, and flexibility of deliverables. The reliability of an item is evaluated by analyzing the loading of the item on its construction. The reliability of the measures was analyzed in this study based on the Cronbach's alpha coefficient. To ensure that the measurements represent all relevant characteristics of their constructs, the content validity of measuring instruments was further verified by experts, individuals knowledgeable about the ICT field and KM implementation in the public sector, to ensure that the measures contained a sufficient and representative set of things fitting into the concepts. The expert analyzed the content validity based on its relevancy as an instrument and questioned whether it was sufficiently accurate to evaluate what this research expects to achieve. The determinants of KMS success in the public sector are outlined in Appendix 1.

Ethical approval for this research experiment was obtained from the UNITEN Ethical Committee at the College of Graduate Studies. The research experiment titled “Determinants of KM Systems Success in Public Sector: A Case of Malaysian Public Sectors” was granted ethical approval with the reference number (2021/07/324) on July 22, 2021. The UNITEN Ethical Committee carefully evaluated the ethical considerations presented in the application and found that the proposed study meets the required ethical standards.

Informed consent was obtained from all participants involved in the study to ensure their voluntary participation and to secure their rights and welfare. The participants were provided with detailed information about the research before their consent was obtained.

### 4.2 Survey distribution and collection

This study used an online survey via email and other social media (e.g., WhatsApp, Telegram, Facebook) to collect data for investigating the hypotheses. For this study, the respondents to the questionnaire survey (Google Form) were employees of the Malaysian Public Sectors (MPS) in different schemes and grades in the respective government departments and ministries in Malaysia. To participate in this survey, the respondents were expected to use any of the ICT tools for sharing and seeking knowledge within public sector organizations. A total of 162 responses were collected by the end of September 2021 after two follow-ups. Out of 162 questionnaires received, four responses were rejected (e.g. redundancy of responses) and were excluded from the sample, leaving 158 valid responses. All the completed questionnaires were evaluated for the data analysis by using SmartPLS. A summary of the questionnaire survey results is outlined in Table 1.

**Table 1:** Summary of questionnaire survey.

Results	Number of Questionnaires
Responses accepted after first disseminated	90
Responses accepted after first reminder	42
Response accepted after second reminder	30
Total rejected (redundancy of responses)	4
Total useable returned questionnaires	158

### 4.3 Data analysis

We employed PLS-SEM to analyze the collected data. PLS-SEM is suitable for exploratory research like ours, as it can effectively handle complex models and smaller sample sizes. This method allowed us to assess the relationships between the variables in our model and identify the factors with the most significant impact on KMS success. We selected PLS-SEM for the following reasons:

(1) Suitability to our research context: PLS-SEM is well-suited for exploratory research, particularly when dealing with complex models and small sample sizes. Given the unique context of our study within the public sector, which often involves limited resources and complex interrelationships, PLS-SEM provides a flexible and robust approach to model assessment.
(2) Predictive focus: PLS-SEM is known for its predictive capabilities. Since our primary goal is to identify factors influencing KMS success and their relationships, we value the predictive power of PLS-SEM to help us understand which factors have the most significant impact on KMS use.

### 4.4 Hypothesis testing

In our study, we formulated and tested hypotheses to validate the relationships between variables. These hypotheses were derived from our conceptual framework and previous research in the field.

## 5 Results

The PLS-SEM technique is used in this study to verify the model, which includes the following steps: (1) construct measurement analysis and (2) determining the relationship between the constructs. The measurement model was validated using the output of the PLS algorithm applied to the survey data. During data analysis, the reliability of each item, the consistency and reliability of each construct's measure, and the convergent and discriminant validity were all checked. Meanwhile, the statistical significance of path coefficients was estimated using the bootstrap resampling method. The data analysis results, including the respondents' demographics, are also presented in this section.

### 5.1 Descriptive analysis

The questionnaire survey received 158 valid (N) responses, representing the total number of employees in the federal government (27 ministries), semi-government located in Wilayah Persekutuan Putrajaya and Klang Valley, and state government that have utilized KMS within the organization. The frequencies and percentages of the profiles of the respondents are presented in Table 2.

**Table 2:** Profile of respondents (N = 158).

Demographic attribute	Category	Frequency	%
Gender	Male Female	89 69	56.3 43.7
Age (years)	18–30 31–40 41–50 51 and above	8 89 50 11	5.1 56.3 31.6 7.0
Agency	Federal Government Semi-government State government	146 5 7	92.4 3.2 4.4
No. Of employees	1–50 51–100 101–200 201–300 301–400 Above 400	21 49 21 7 6 54	13.3 31.0 13.3 4.4 3.8 34.2
Years of computer experience	1–5 6–10 11–15 Above 15	5 16 40 97	3.2 10.1 25.3 61.4

**Fig. 2:**
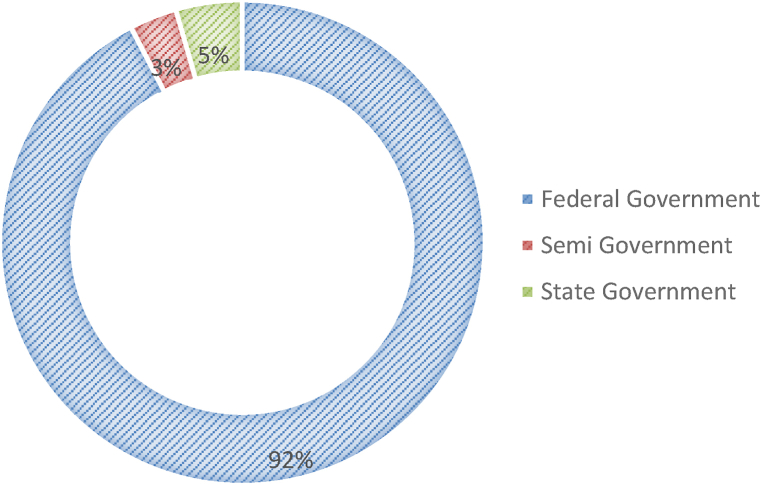
Respondents from government agencies.

Fig. 2 indicates the types of government agencies in which the respondents worked in the public sector. Employees with various schemes and grades from the federal government (92.4%), followed by the state government (4.4%) and semi-government (3.2%), were among those who responded to this survey. Because the number of federal government employees in MPS is greater than that of other government agencies, this suggests that the number of respondents in federal government agencies is higher than that in state and semi-government agencies.

The results show that the respondents worked for 17 of the 26 federal government organizations (89.8%) and other organizations (10.2%), which represent state government and semi-government organizations. Kementerian Tenaga dan Sumber Asli Malaysia respondents were the highest (32.4%) for the federal government, consisting of several divisions and departments that have implemented KMS in their organizations.

**Fig. 3:**
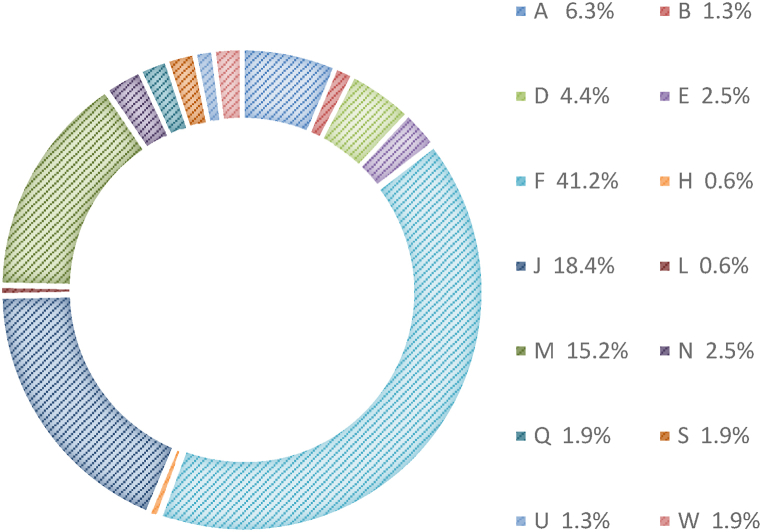
Respondents by job scheme.

Fig. 3 presents the respondents in government agencies according to their job schemes. The respondents were employees in the public sector with various schemes and grades. The chart shows that the F scheme (information technology) had the highest respondents (41.2%), followed by the J scheme (Engineering) as the second highest respondents (18.4%), and the M scheme (Administration and Diplomatic) as the third highest respondents (15.2%). These findings suggest that knowledge sharing by employees with various job schemes significantly influences the success of KMS implementation in public sector organizations.

**Fig. 4:**
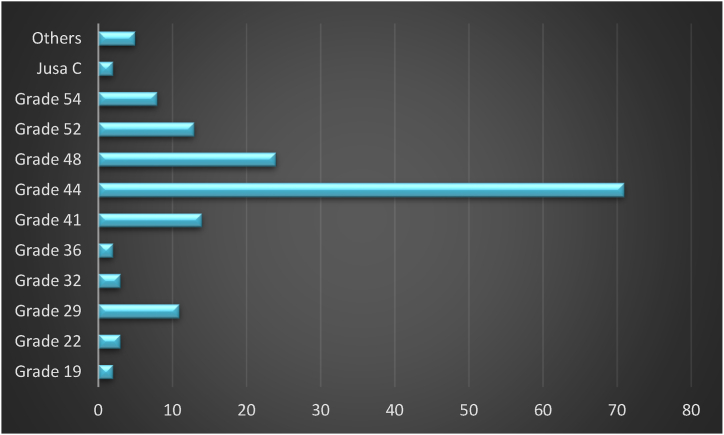
Respondents by job grades (position).

Fig. 4 presents the respondents in government agencies according to their job grades or positions. From the chart above, three major schemes of service groups responded to the questionnaire: (1) top management group including grade JUSA C (vice-secretary general of ministry) and grade 54 (director of the department/division) with 6.4% of respondents, which are the policy-makers in an organization; (2) management and professional group including grade 52 (deputy director), grade 48 (chief assistant director), grade 44 (senior assistant director), and grade 41 (assistant director) with 77.2% of respondents; and (3) support group including grade 36 (the highest senior assistant officer), grade 32 (senior assistant officer), grade 29 (assistant officer), grade 22 (senior support staff), and grade 19 (support staff) with 13.4% of respondents. This suggests that management and professional groups with a secondary hierarchy in the Malaysian public sector were the highest respondents to the study.

### 5.2 Assessment of the measurement model

Using Cronbach's alpha and composite reliability, the constructs' internal consistency was evaluated. SmartPLS advises observing and reporting on both internal consistency criteria. In exploratory research, an acceptable internal consistency range is 0.60–0.70 [43]. AVE (average variance extracted) and outer loadings were used to assess the convergent validity. Table 3 presents the constructs' convergent validity and reliability.

**Table 3:** Psychometric properties of the constructs (N = 158).

Construct	Reliability		Convergent validity		
	Cronbach α	Composite			
		Reliability	K	Loading range	AVE
Knowledge content quality	0.94	0.93	7	0.75–0.84	0.65
KM system quality	0.96	0.94	4	0.88–0.92	0.80
Perceived usefulness of KMS	0.95	0.96	4	0.91–0.94	0.85
User satisfaction	0.95	0.96	5	0.89–0.93	0.82
Culture of sharing	0.95	0.96	5	0.89–0.93	0.83
Perceived trust	0.94	0.97	3	0.94–0.96	0.90
Leadership	0.95	0.95	5	0.87–0.91	0.81
Incentive	0.92	0.94	5	0.82–0.91	0.76
KMS use	0.91	0.97	5	0.88–0.95	0.85

Note: K = number of indicators; AVE = average variance extracted.

All of the scales complied with the criteria for internal consistency, as evidenced by the Cronbach's alpha and composite reliability scores in the aforementioned table. The values for AVE were in the range between 0.65 and 0.90, demonstrating the scales' strong convergent validity. Hair (2021) [43], recommends an AVE of 0.5 or above.

To determine the discriminant validity of the constructs, the heterotrait–monotrait ratio of correlations (HTMT) criterion was applied. Henseler et al. (2015) [44], claimed that if the HTMT value between two constructs is less than 0.90, discriminant validity has been demonstrated. Table 4 displays the constructs’ discriminant validity.

**Table 4:** Discriminant validity of the constructs (N = 158).

		1	2	3	4	5	6	7	8	9
1	Knowledge content quality	–								
2	KM system quality	0.82	–							
3	Perceived usefulness of KMS	0.81	0.79	–						
4	User satisfaction	0.86	0.77	0.83	–					
5	Culture of sharing	0.64	0.63	0.61	0.65	–				
6	Perceived trust	0.70	0.62	0.62	0.63	0.65	–			
7	Leadership	0.66	0.66	0.62	0.65	0.84	0.61	–		
8	Incentive	0.41	0.31	0.25	0.34	0.54	0.52	0.50	–	
9	KMS use	0.75	0.70	0.78	0.79	0.61	0.65	0.68	0.32	–

### 5.3 Assessment of the structural model

To determine multicollinearity, the correlation between independent variables of the model was examined. It was discovered using the VIF statistic. The value of VIF should be less than 5 [43]. Collinearity statistics of the constructs are presented in Table 5, where the VIF value for exogenous constructs corresponding to all endogenous constructs is less than 4. There is no correlation between exogenous constructs. The assumption of multicollinearity is fulfilled.

**Table 5:** Collinearity statistics (VIF) of the constructs (N = 158).

	Knowledge content quality	Perceived usefulness of KMS	User satisfaction	KMS use
Knowledge content quality		2.78	2.83	
KM system quality		2.50	2.75	
Perceived usefulness of KMS			2.76	2.75
User satisfaction				2.90
Culture of sharing		1.90		
Perceived trust		2.06		
Leadership	1.28			1.68
Incentive	1.28			
KMS use				

The coefficient of determination (R^2^) and prediction relevance (R^2^) were used to evaluate the model's predictive ability. The bootstrapping method was used to ascertain the path coefficients' statistical significance. For linked endogenous constructs, the R^2^ coefficient of all external constructs was examined. Table 6 displays the constructs' coefficients of determination and predictive relevance.

**Table 6:** Coefficient of determination and predictive relevance of the study constructs (N = 158).

Construct	R^2^	Q^2^
Knowledge content quality	0.38	0.22
Perceived usefulness of KMS	0.65	0.51
User satisfaction	0.73	0.55
KMS use	0.63	0.51

Note: R^2^ = coefficient of determination; Q^2^ = predictive relevance.

All independent variables are moderate to strong predictors of dependent variables, according to the values shown in the above table. R^2^ and Q^2^ values of 0.25 can be regarded as weak, 0.50 as moderate, and 0.75 as strong, according to Hair et al. (2021) [43]. Knowledge content quality was directly influenced by leadership and had a much weaker predictive relevance; however, it is weak to moderate in sample predictive power. The model appears to be good overall based on the amount of variance explained for dependent variables.

### 5.4 Hypothesis testing

The path coefficient analysis and the construct results are presented in Table 7. The findings, including hypotheses H1, H2, H3, H5, H6, H7, H10, and H11, supported eight hypotheses. Meanwhile, four hypotheses were not supported, including hypotheses H4, H8, H9, and H12.

**Table 7:** Structural model assessments (N = 158).

H#	Path	Path coefficient β	p-Value	t-Value	f^2^	Results
1	Knowledge content quality→perceived usefulness of KMS	0.37	0.00	4.04	0.14	Supported***
2	Knowledge content quality→user satisfaction	0.43	0.00	5.02	0.24	Supported***
3	KM system quality→perceived usefulness of KMS	0.36	0.00	3.86	0.15	Supported***
4	KM system quality→user satisfaction	0.12	0.15	1.44	0.02	Not supported
5	Perceived usefulness of KMS→KMS use	0.32	0.00	4.14	0.11	Supported***
6	Perceived usefulness of KMS→user satisfaction	0.38	0.00	4.80	0.19	Supported***
7	User satisfaction→KMS use	0.36	0.00	3.30	0.13	Supported**
8	Culture of sharing→perceived usefulness of KMS	0.11	0.28	1.08	0.02	Not supported
9	Perceived trust→perceived usefulness of KMS	0.07	0.38	0.89	0.01	Not supported
10	Leadership→knowledge content quality	0.55	0.00	7.46	0.38	Supported***
11	Leadership→KMS use	0.24	0.01	2.61	0.10	Supported*
12	Incentive→knowledge content quality	0.13	0.06	1.89	0.02	Not supported

The results of the system factors show that knowledge content quality significantly affects the perceived usefulness of KMS with a small effect size (f^2^ = 0.14) and has a significant effect on user satisfaction with a medium effect size (f^2^ = 0.24). Thus, the results of hypotheses H1 and H2 were supported. KM system quality has a significant effect on the perceived usefulness of KMS with a medium effect size (f^2^ = 0.15); therefore, the result of hypothesis H3 was supported. However, the current study found no statistically significant relationship between KM system quality and user satisfaction (p = 0.15). Therefore, hypothesis H4 was not supported. The perceived usefulness of KMS has a significant effect on KMS use with a small effect size (f^2^ = 0.11) and a significant effect on user satisfaction with a medium effect size (f^2^ = 0.19). Thus, the results of hypotheses H5 and H6 were supported. User satisfaction has a significant effect on KMS use with a small effect size (f2 = 0.13); therefore, the result of hypothesis H7 was supported.

Of the organizational factors, the culture of sharing (p = 0.28) and perceived trust (p = 0.38) found no statistically significant relationship with the perceived usefulness of KMS. Therefore, the results of hypotheses H8 and H9 were not supported. Leadership has a significant effect on knowledge content quality with a large effect size (f^2^ = 0.38) and a significant effect on KMS use with a small effect size (f^2^ = 0.10). Thus, the results of hypotheses H10 and H11 were supported. The current study found no statistically significant relationship between the incentive and knowledge content quality (p = 0.06). Therefore, the result of hypothesis H12 was not supported.

## 6 Discussion

The notion in the finding indicates that the quality of knowledge has an impact on the overall success of KMS implementation. Employees in public sector organizations in Malaysia are willing to use KMS for knowledge sharing, as evidenced by the significant impact of knowledge content quality on the perceived usefulness of KMS and user satisfaction if they find the KMS to be of good quality (e.g., easy to use, user-friendly, efficient) while at the same time finding the KMS useful and relevant to their job performance. Therefore, specific quality and content mechanisms should be established in the operation of the knowledge-sharing platform to avoid any malicious content (e.g., viruses, ransomware) that could disrupt or destroy KMS partially or completely. The results show that the quality of the KM systems influenced the perceived usefulness of KMS. However, it was not found to influence user satisfaction significantly. This indicates that the employees perceive KMS as useful for them if the quality of the systems is easy to use and user-friendly and the response time is acceptable. If the system quality is poor, which requires the employees to overcome many hurdles in using the KMS, they are unlikely to feel that the KMS is beneficial.

It was discovered that user satisfaction was impacted by how useful KMS was deemed to be. Employees in public sector in Malaysia are more likely to use a KMS's services if they believe they will improve job quality and advance their performance goals and those of their organization. While the perceived usefulness of KMS has been identified as consistently important in promoting its use, support for user satisfaction has been inconsistent and of less significance. This is because whether the system meets user expectations is subjective, depending on each individual's interpretation of satisfaction. As found in this study, the employees were more influenced to use KMS by their satisfaction with the benefits provided by the KMS, which in turn is influenced by the content quality provided in KMS, as compared to their satisfaction level with KMS, which is influenced by KM system quality [[38], [45], [46]].

The findings show that from the organizational perspective, the most significant effect on KMS use is leadership, indicating the importance of leaders in persuading employees to share their knowledge via KMS, which in turn may naturally create a culture of sharing. Therefore, implementing successful KMS does not require a culture of sharing, but it does require good leadership to create the culture, thereby increasing the use of KMS. Trust is critical for KM processes such as providing transparent knowledge or utilizing and sharing knowledge. The utilization of information technology to share knowledge allows for a more efficient and organized working environment, but it can also reveal individuals’ information. In the public sector, where top management is the most authoritative and influential decision-maker, the active participation of leaders as knowledge contributors is critical for the success of KMS. Employees would prefer to see knowledge sharing in action by leaders providing their knowledge rather than having the impression that leaders are dedicated to KMS through policy implementation.

The insignificant effect of incentives on KMS use via knowledge content quality suggests that incentives in the public sector are not as important as in other sectors. Employees do not depend on the incentive mechanism to influence them to contribute good-quality knowledge. Employees are more influenced by their leaders to contribute quality knowledge to KMS, as found in this study. Furthermore, currently, in the public sector, no incentive mechanism is keeping track of contributions by employees on a communication platform such as emails or social media established in public sector organizations. One possible reason for the insignificant effect of incentives in the current study is the low incentives provided to employees for knowledge sharing. Knowledge sharing via email, social media, or video conferencing through informal ICT platforms is more practiced among employees, which cannot be measured accurately and thus cannot be expected for any monetary incentives.

Regarding limitations of this study, it is emphasized that while inspiration is drawn from the well-established DeLone and McLean model, the proposed model has been intentionally adapted to address the distinctive context of the public sector in Malaysia. The modification takes into account the specific challenges and intricacies associated with implementing KMS in this particular setting. It is crucial to recognize that the model is not a direct replication but rather an integrated model tailored aimed at capturing the nuanced factors influencing KMS success in Malaysian public sector organizations.

## 7 Implications

For research, a primary contribution to the research community in KMS is the integration of organizational factors with the theory of IS success by DeLone and McLean, who initially emphasized the technical aspects of the information systems and perceptions of usefulness and user satisfaction. This study adds to the KM literature on the theoretical aspect of KM that emphasizes the interactions of people, technology, and process to establish KM, and hence organizational factors are important elements in KM. More specifically, this study consider specific organizational factors (leadership, perceived trust, and culture of sharing) that may or may not influence the success of KMS. By including organizational factors in the success model, this study provides a deeper understanding of the broader context of the success of information systems, in particular KMS, which adds to the IS success literature.

For practice, top management should lead by example to advocate knowledge sharing, use KMS to contribute ideas, and cultivate employees’ interest in knowledge-sharing activities. The public sector, through its leaders, should create clear organizational goals and support change management to promote employee involvement and contribution.

The public sector should create a solid knowledge content review system that governs the content creation process and ensures the quality and competence of the information presented. Employee assessment mechanisms should be closely followed to regularly assess perceived usefulness and user satisfaction, and any suggestions should be given substantial implications.

Furthermore, this study's findings highlight the significance of establishing a capable KMS by providing employees with dependable and consistent system operations, high-quality knowledge, and a user-friendly environment. A well-designed KMS can improve employee satisfaction by reducing potential uncertainties and risks associated with concerns about the dependability and usefulness of the KMS that supports knowledge-sharing activities. This may aid in developing and maintaining favorable attitudes toward the success of KMS among employees, which contribute to higher motivation to continue using KMS.

Finally, public sector leadership tries to strengthen decision-making processes since public sector decisions rely on the availability of high-quality knowledge. Leadership that promotes high-quality achievements in KM systems is likely to be very effective.

## 8 Conclusion

Public sector organizations have good opportunities to expand their use of KMS to be more effective, as KM is regarded as a competitive advantage. As a result, recognizing the factors influencing KMS's success is critical to ensuring a successful implementation strategy. The objective of this study was to determine system and organizational factors affecting KMS success in the public sector by focusing on KMS use as the measure of success. KMS use by employees means the use of KMS for sharing knowledge. A conceptual model was developed based on previous studies of the KMS success model and the theory of IS success by DeLone and McLean. A cross-sectional survey was conducted to test the model developed in this study. Participants in the study were employees of Malaysia's public sector. The conceptual model was tested using the PLS-SEM approach.

Among the system factors indicated, knowledge content quality was discovered to influence KMS use via the perceived usefulness of KMS and user satisfaction. Meanwhile, the perceived usefulness of KMS has been found to impact KM system quality. Of the organizational factors identified, leadership had a significant effect on both knowledge content quality and KMS use. Knowledge content quality is a system factor that has the most significant impact on KMS use. At the same time, leadership is the organizational factor with the greatest overall impact on KMS use. Twelve hypotheses were included to be tested in this study, resulting in eight hypotheses being supported.

Knowledge content quality was found to be a strong predictor of KMS success; therefore, increasing knowledge content quality is essential for maintaining the successful implementation of KMS in Malaysia's public sectors. Leadership was the most significant effect of organizational factors. Top management should perform important responsibilities with respect to leadership style to improve the quality of knowledge content. Therefore, employees will strive to share knowledge with each other, thus generating a high level of sharing and dissemination of information.

This study proposed a conceptual model of KMS success in the public sector that outlined the factors contributing to the success of KMS implementation in the public sector. The results can be a reference for public sector management interested in implementing a successful KMS. Future research on the success of KMS implementation in other public sector organizations must evaluate the factors found and analyze the relationships between them.

## Data availability statement

Data can be requested from the corresponding author.

## CRediT authorship contribution statement

**Nor'ashikin Alia:** Writing – original draft, Supervision, Methodology, Conceptualization. **Musyazid Md Mustaffa:** Writing – original draft, Visualization, Methodology, Investigation, Data curation, Conceptualization. **Gamal Alkawsi:** Writing – review & editing, Visualization, Formal analysis. **Luiz Fernando Capretz:** Writing – review & editing, Supervision.

## Declaration of competing interest

The authors declare the following financial interests/personal relationships which may be considered as potential competing interests: Norshikin Bte Ali reports financial support was provided by 10.13039/501100000038NSERC ( Natural Sciences and Engineering Research Council of Canada) Discovery Grant Number: RGPIN-2020-04325.
