# Supplementary figures and images for: If you build it, will they come? Exploring the success factors of knowledge management systems in the Malaysian public sector
Source: Heliyon. 2024 Mar 7;10(6):e27093. doi: 10.1016/j.heliyon.2024.e27093 (PMC10950497; doi:10.1016/j.heliyon.2024.e27093)

**
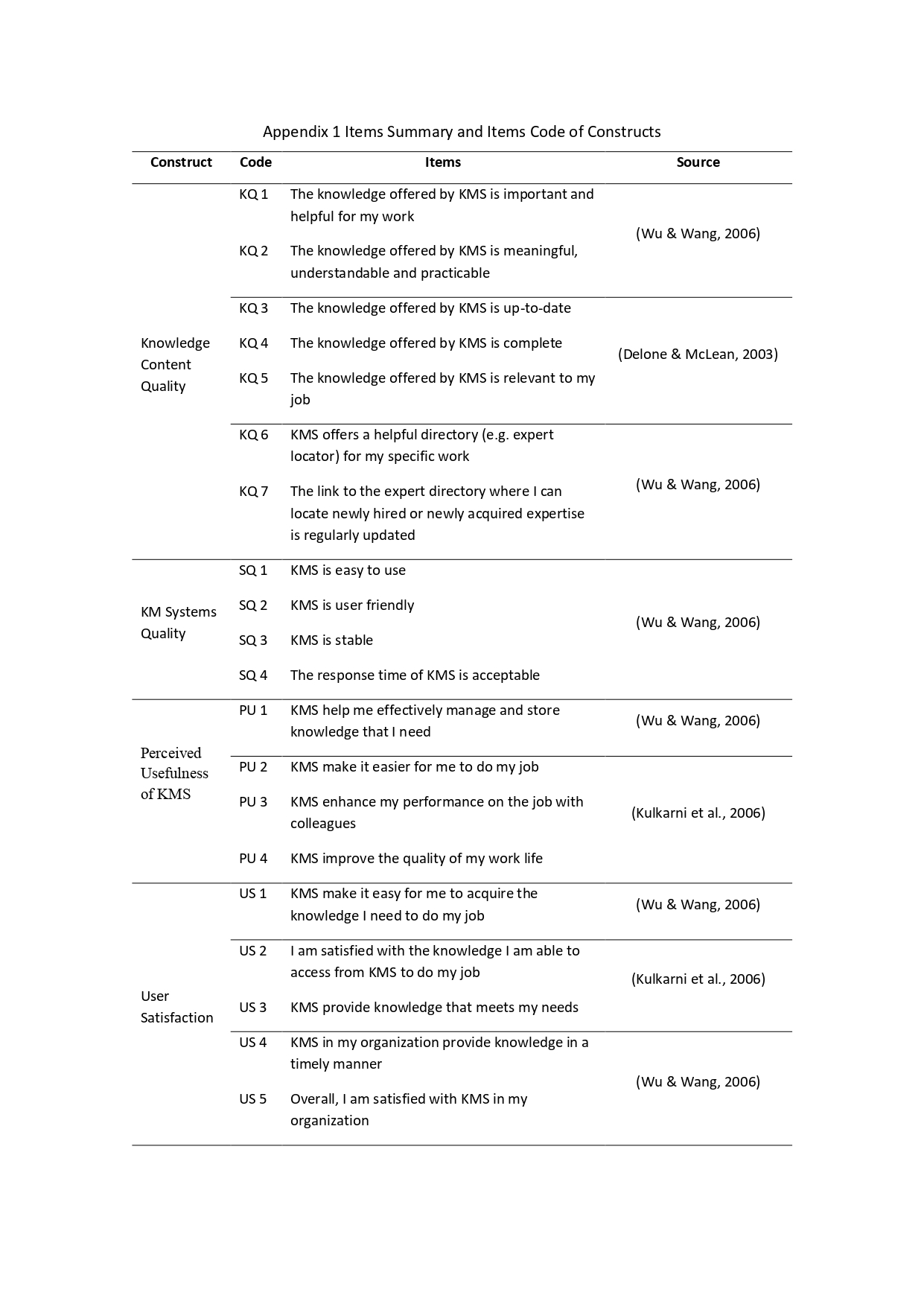

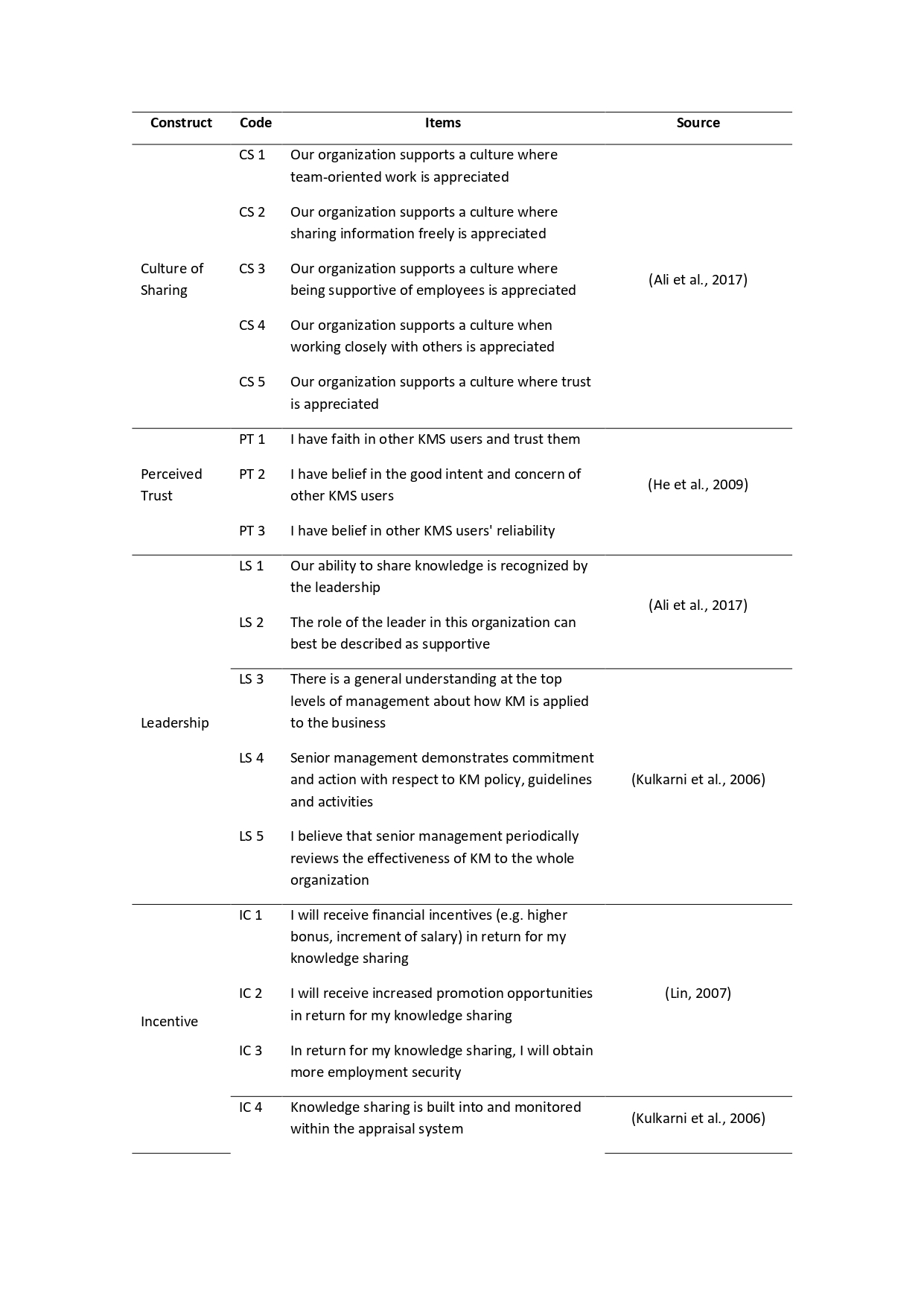

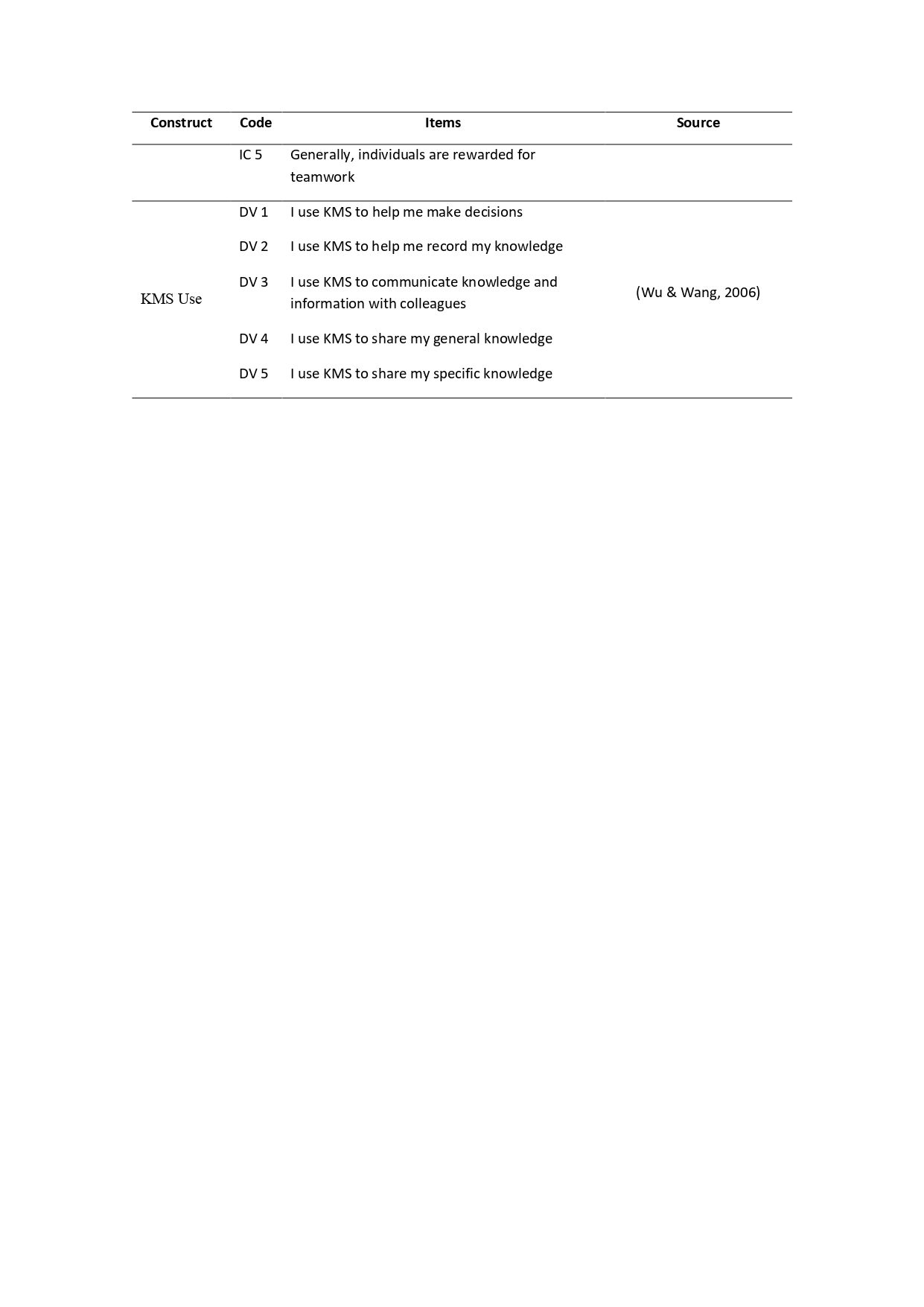
**

Supplement: Multimedia component 1 [file mmc1.docx]
